# Supplementary material for: Ecological constraints and evolutionary trade-offs shape nitrogen fixation across habitats
Source: ISME Commun. 2026 Jan 15;6(1):ycag007. doi: 10.1093/ismeco/ycag007 (PMC12903952; doi:10.1093/ismeco/ycag007)
Supplement: Sobol_ISME-Comm_2026_SI_figures_ycag007 [file sobol_isme-comm_2026_si_figures_ycag007.docx]

Supporting Information

Ecological constraints and evolutionary trade-offs shape nitrogen fixation across habitats

Morgan S. Sobol^1,2*^, Aya S. Klos^1^, Cécile Ané^3,4,^ Katherine D. McMahon^5^ and Betül Kaçar^1*^

1. Department of Bacteriology, University of Wisconsin-Madison, Madison, WI, USA
2. Department of Biology, Texas State University, San Marcos, TX, USA
3. Department of Statistics, University of Wisconsin-Madison, Madison, WI, USA
4. Department of Botany, University of Wisconsin-Madison, Madison, WI, USA
5. Department of Civil and Environmental Engineering, University of Wisconsin–Madison, Madison, WI, USA

**Correspondence:**

Morgan Sobol, Department of Biology, Texas State University, 601 University Drive, San Marcos, TX, 78666, USA, Email: msobol@txstate.edu; Betül Kaçar, Department of Bacteriology, University of Wisconsin-Madison 1550 Linden Drive, Madison, WI, 53706, USA, Email: bkacar@wisc.edu

**Running title:** Evolutionary trade-offs in N_2_ fixation

**
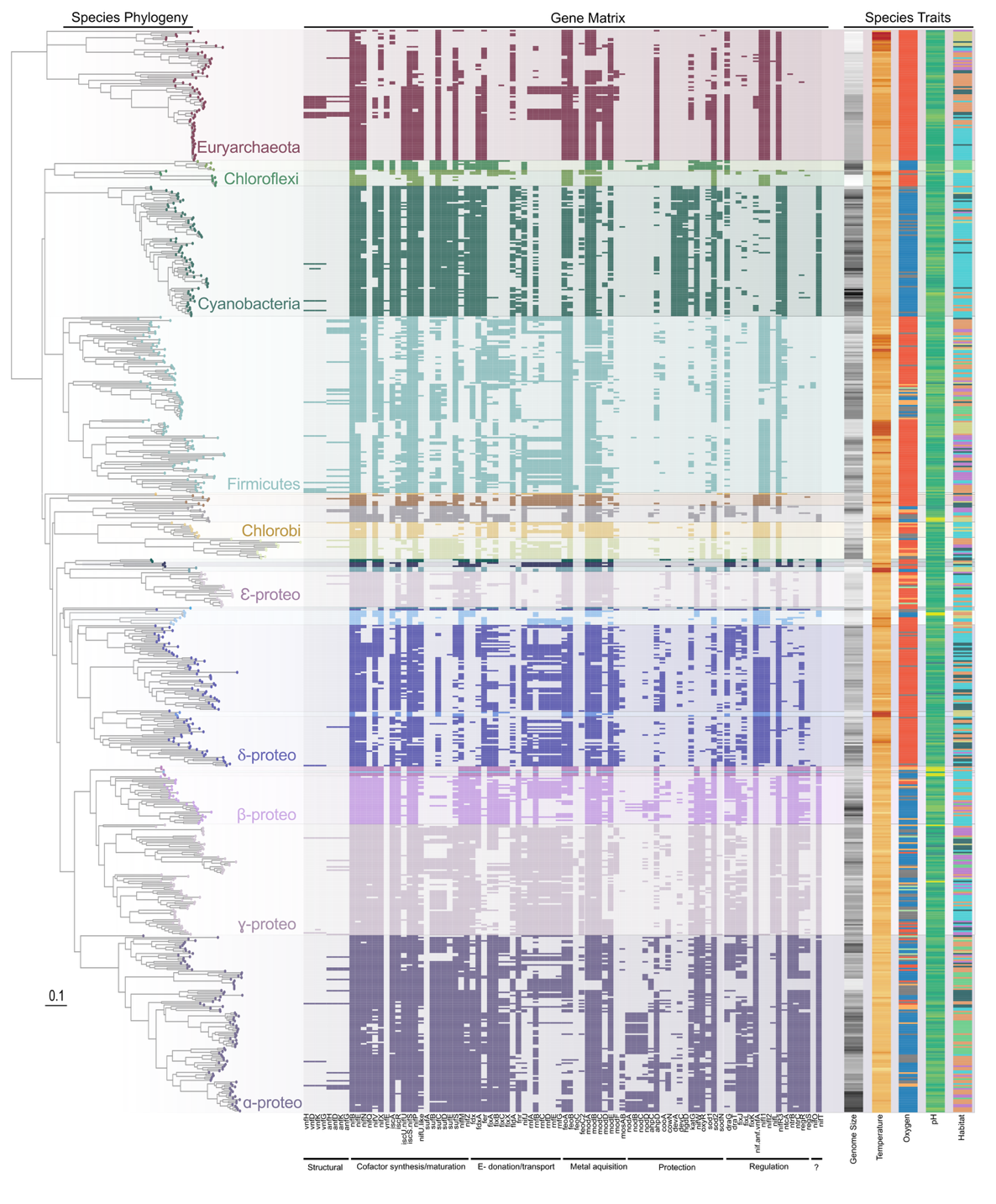
**

**Supplemental Figure 1.** Presence-absence distribution N_2_ fixation genes amongst genomes that have nitrogenase in the Zhu et al (2020) Web of Life (WoL) phylogeny [1]. This figure accompanies Figure 1 in the main manuscript. Genes are organized into categories based on their functional role (structural, cofactor synthesis, electron donation/transport, metal acquisition, protection, regulation, and unknown). The “?” indicates genes *nifO* and *nifT* whose functions are unknown. The gene presence-absence matrix was determined via homology search with HMMER against the KEGG KO database [2]. Species-associated ecological and physiological traits were mapped alongside the phylogeny and matrix to highlight associations in genes and gene abundance with different lifestyles. The list of genes and their roles can be found in Supplemental Table 1, and the gene matrix and associated categories can be found in Supplemental Table 3. The phylogeny and matrices were plotted using the ggTree R package [3].

**
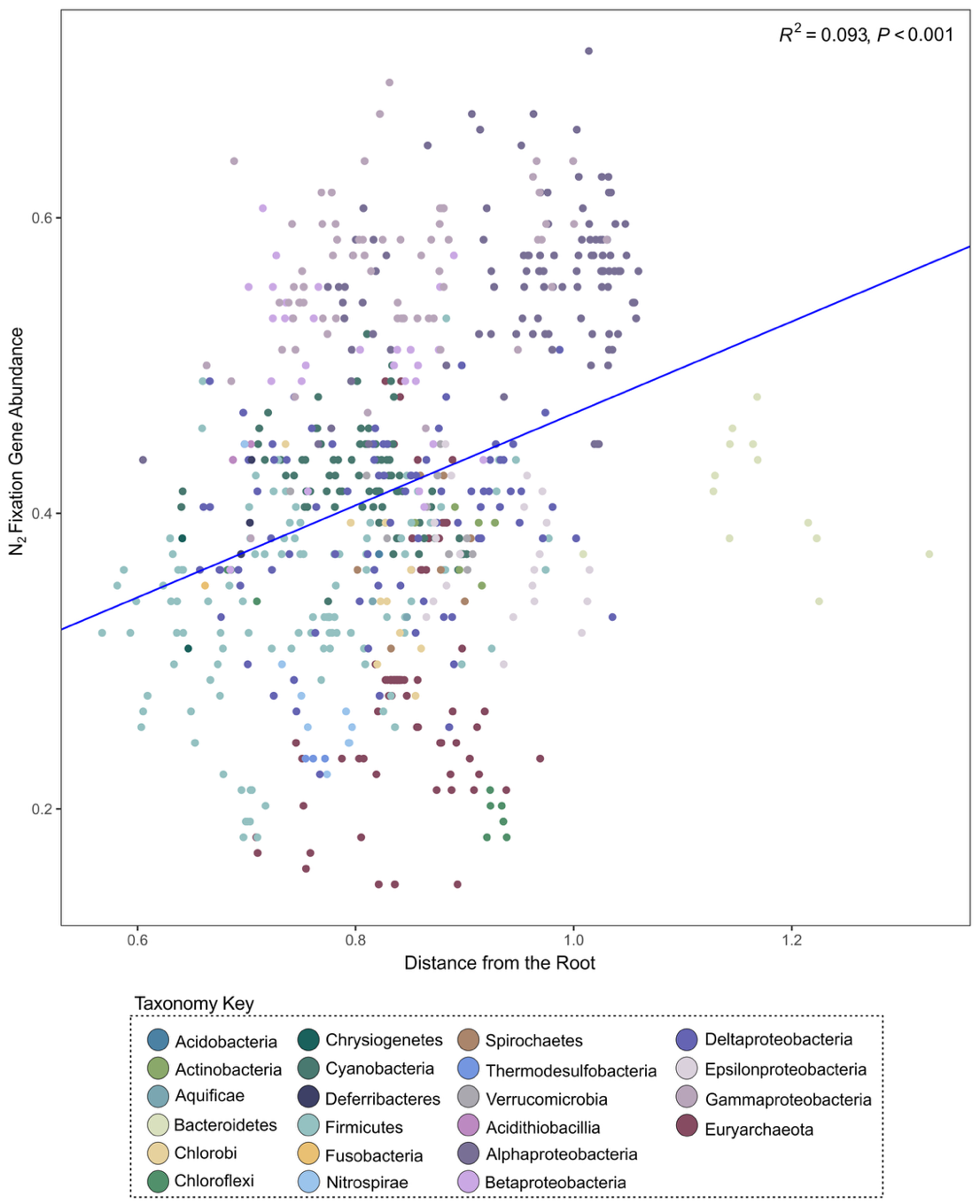
**

**Supplemental Figure 2.** Relationship between patristic distances and N_2_ fixation gene abundance. A positive relationship was observed, indicating that lineages with greater cumulative evolutionary change tend to have larger N_2_ fixation gene repertoires (*P*<0.001). Each point represents a genome, colored by taxonomic affiliation. The linear regression and trendline were calculated using the *lm* function in the stats R package [4]. The *distRoot* function in the **adephylo** R package [5] was used to calculate the root-to-tip patristic distances. The scatter plot was plotted using ggplot2 in R [6].

**Supplemental Figure 3. Oxygen status, optimal growth temperature, and optimal pH of diazotroph and non-diazotroph-associated genomes.** (a) Bar plots show the proportion of genomes associated with aerobes, anaerobes, facultative anaerobes, or microaerophiles for diazotrophs (n=673) and non-diazotrophs (n=700), respectively. Gray represents taxa whose oxygen status could not be classified due to missing data or uncertainty in predictions. A literature search was performed to verify oxygen status predictions and supplement information for diazotrophs, resulting in a smaller proportion of taxa with missing information. This was done to a lesser extent for non-diazotrophs with references shown in Supplemental Table 9. Boxplots show the distribution of optimal growth temperature (OGT) (b) and pH (c) for diazotrophs and non-diazotrophs. Wilcoxon rank-sum tests were used to compare the medians of quantitative trait variables (OGT and pH) between diazotrophs and non-diazotrophs. The distribution of temperatures differs between both groups where diazotrophs have a higher median OGT (*P*-value=1.35×10^-6^), while no statistical difference was detected between the distributions of pHs for diazotrophs vs non-diazotrophs (*P*-value=0.0750). Statistical tests were performed using functions in base R [4], and plots were made using ggplot2 [6].

**Supplemental Figure 4.** Phylogenetic linear regressions assessing whether the richness of N_2_ fixation-related genes can predict different traits of diazotroph hosts. The different traits assessed are shown on the y-axes and include genome size (A), temperature (B), metabolic coverage (C), and habitat coverage (D). Response variables were either transformed using natural log or square root to stabilize variance prior analysis. Solid blue lines represent non-phylogenetic linear models, dashed blue lines represent phylogenetic linear models. Correlation coefficients (*R^2^*) and associated *P*-values for both regression models, and lambda values are shown for each plot. Scatter plots were plotted using ggplot2 in R [6]. Points are colored by taxonomy following the color scheme in the legend of Figure 5 in the main text. The same regressions were performed to predict optimal growth temperature, metabolic, and habitat coverage after normalizing gene richness to genome size and are shown in Figure 5 of the main text. Note that (a) is identical to Figure 5A of the main text and is shown here for comparison purposes.

**Supplemental Figure 5.** Phylogenetic linear regressions assessing whether the richness of N_2_ fixation-related genes can predict different traits of non-diazotroph hosts. The different traits assessed are shown on the y-axes and include genome size (A), temperature (B), metabolic coverage (C), and habitat coverage (D). Response variables were either transformed using natural log or square root to stabilize variance prior analysis. Solid blue lines represent non-phylogenetic linear models, dashed blue lines represent phylogenetic linear models. Correlation coefficients (*R^2^*) and associated *P*-values for both regression models, and lambda values are shown for each plot. Scatter plots were plotted using ggplot2 in R [6]. Points are colored by taxonomy following the color scheme in the legend of Figure 5 in the main text.

**Supplementary Figure 6.** Phylogenetic linear regressions assessing whether the richness of N_2_-fixation genes belonging to different functional groups can predict the size of genomes of diazotrophs. Functional groups include those related to nitrogenase structure (A), cofactor synthesis/maturation (B), electron donation/transport (C), metal acquisition (D), protection (E), and regulation (F). Response variables were either transformed using natural log or square root to stabilize variance prior analysis. Solid blue lines represent non-phylogenetic linear models, dashed blue lines represent phylogenetic linear models. Correlation coefficients (*R^2^*) and associated *P*-values for both regression models, and lambda values are shown for each plot. Scatter plots were plotted using ggplot2 in R [6]. Points are colored by taxonomy following the color scheme in the legend of Figure 5 in the main text.

**Supplementary Figure 7.** Phylogenetic linear regressions assessing whether the richness of N_2_-fixation genes belonging to different functional groups can predict the size of genomes of non-diazotrophs. Functional groups include those related to nitrogenase structure (A), cofactor synthesis/maturation (B), electron donation/transport (C), metal acquisition (D), protection (E), and regulation (F). Response variables were either transformed using natural log or square root to stabilize variance prior analysis. Solid blue lines represent non-phylogenetic linear models, dashed blue lines represent phylogenetic linear models. Correlation coefficients (*R^2^*) and associated *P*-values for both regression models, and lambda values are shown for each plot. Scatter plots were plotted using ggplot2 in R [6]. Points are colored by taxonomy following the color scheme in the legend of Figure 5 in the main text. All correlations for phylogenetic regressions are notably weak, suggesting phylogenetic relationships may be an important factor driving the observed trends between traits and gene richness. One exception is the correlation between genome size and protection gene richness which shared a positive correlation (*R^2^*=0.137, *P*=4.67×10^-21^), suggesting oxygen protection genes may have been more involved in genome expansions/subtractions in non-diazotrophs, compared to genes from other functional groups.

**Supplementary Figure 8.** Phylogenetic linear regressions assessing whether the richness of N_2_-fixation genes belonging to different functional groups can predict metabolic coverage of diazotrophs. Functional groups include those related to nitrogenase structure (A), cofactor synthesis/maturation (B), electron donation/transport (C), metal acquisition (D), protection (E), and regulation (F). Response variables were either transformed using natural log or square root to stabilize variance prior analysis. Solid blue lines represent non-phylogenetic linear models, dashed blue lines represent phylogenetic linear models. Correlation coefficients (*R^2^*) and associated *P*-values for both regression models, and lambda values are shown for each plot. Scatter plots were plotted using ggplot2 in R [6]. Points are colored by taxonomy following the color scheme in the legend of Figure 5 in the main text.

**Supplementary Figure 9.** Phylogenetic linear regressions assessing whether the richness of N_2_-fixation genes belonging to different functional groups can predict metabolic coverage of non-diazotrophs. Functional groups include those related to nitrogenase structure (A), cofactor synthesis/maturation (B), electron donation/transport (C), metal acquisition (D), protection (E), and regulation (F). Response variables were either transformed using natural log or square root to stabilize variance prior analysis. Solid blue lines represent non-phylogenetic linear models, dashed blue lines represent phylogenetic linear models. Correlation coefficients (*R^2^*) and associated *P*-values for both regression models, and lambda values are shown for each plot. Scatter plots were plotted using ggplot2 in R [6]. Points are colored by taxonomy following the color scheme in the legend of Figure 5 in the main text.

**Supplementary Figure 10.** Phylogenetic linear regressions assessing whether the richness of N_2_-fixation genes belonging to different functional groups can predict habitat coverage of diazotrophs. Functional groups include those related to nitrogenase structure (A), cofactor synthesis/maturation (B), electron donation/transport (C), metal acquisition (D), protection (E), and regulation (F). Response variables were either transformed using natural log or square root to stabilize variance prior analysis. Solid blue lines represent non-phylogenetic linear models, dashed blue lines represent phylogenetic linear models. Correlation coefficients (*R^2^*) and associated *P*-values for both regression models, and lambda values are shown for each plot. Scatter plots were plotted using ggplot2 in R [6]. Points are colored by taxonomy following the color scheme in the legend of Figure 5 in the main text.

**Supplementary Figure 11.** Phylogenetic linear regressions assessing whether the richness of N_2_-fixation genes belonging to different functional groups can predict habitat coverage of non-diazotrophs. Functional groups include those related to nitrogenase structure (A), cofactor synthesis/maturation (B), electron donation/transport (C), metal acquisition (D), protection (E), and regulation (F). Response variables were either transformed using natural log or square root to stabilize variance prior analysis. Solid blue lines represent non-phylogenetic linear models, dashed blue lines represent phylogenetic linear models. Correlation coefficients (*R^2^*) and associated *P*-values for both regression models, and lambda values are shown for each plot. Scatter plots were plotted using ggplot2 in R [6]. Points are colored by taxonomy following the color scheme in the legend of Figure 5 in the main text.


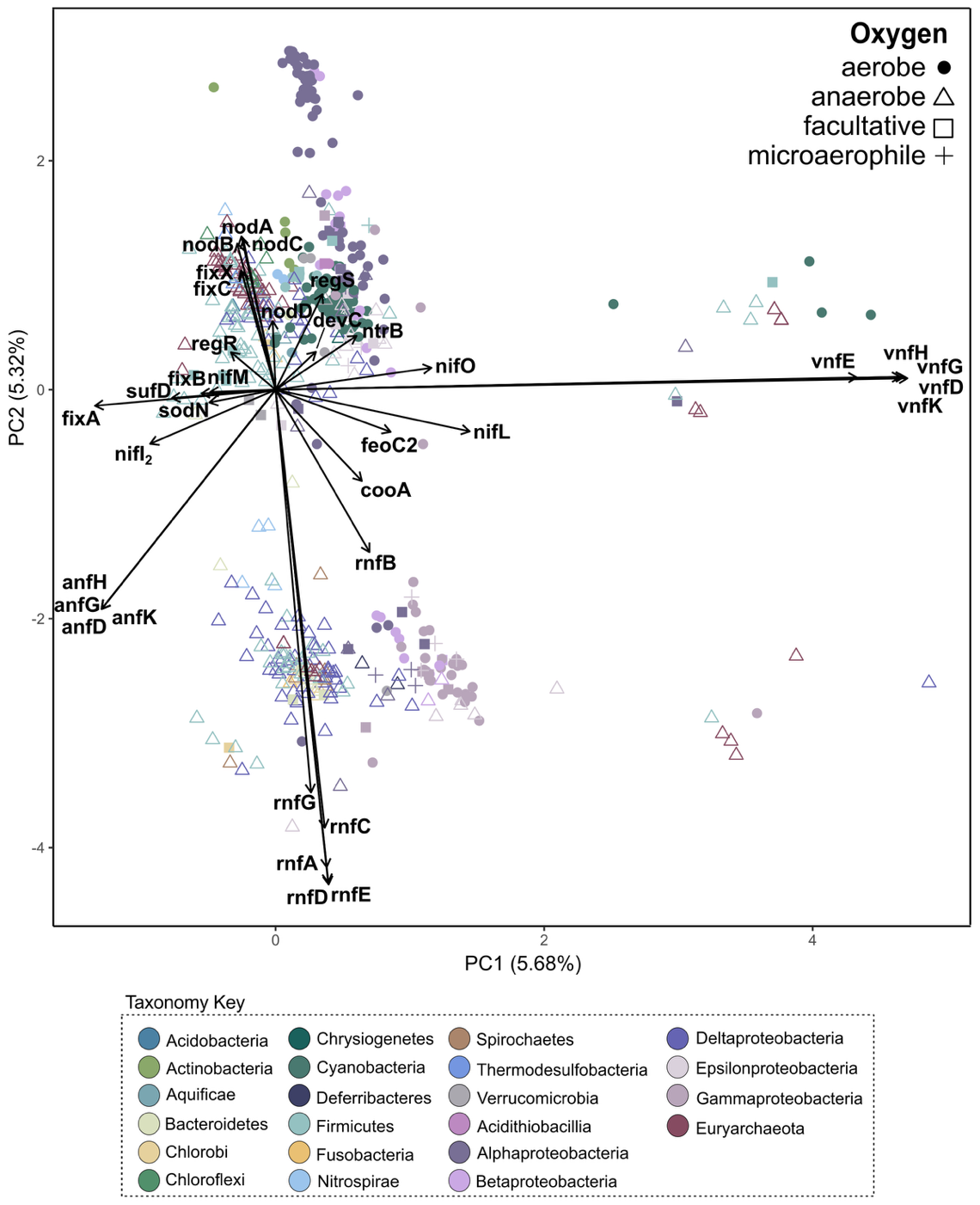


**Supplemental Figure 12.** Phylogenetic principal component analysis (PCA) of genomes clustered by their N_2_ fixation gene presence-absence patterns (Supplemental Figure 1), performed with the *phyl.pca* function (method = “BM”, mode =”corr”) in phytools R package [7]. Each point represents a genome, colored by taxonomic affiliation, and shapes represent oxygen preference. Vectors indicate the top ten genes with the strongest influence on each component. The low explained variance of the first two components (PC1 = 5.68%, PC2 = 5.32%) suggests that most variation is explained by phylogenetic structure such that genomes cluster primarily due to conserved gene sets inherited from common ancestors. The PCA was plotted using ggplot2 in R [6].


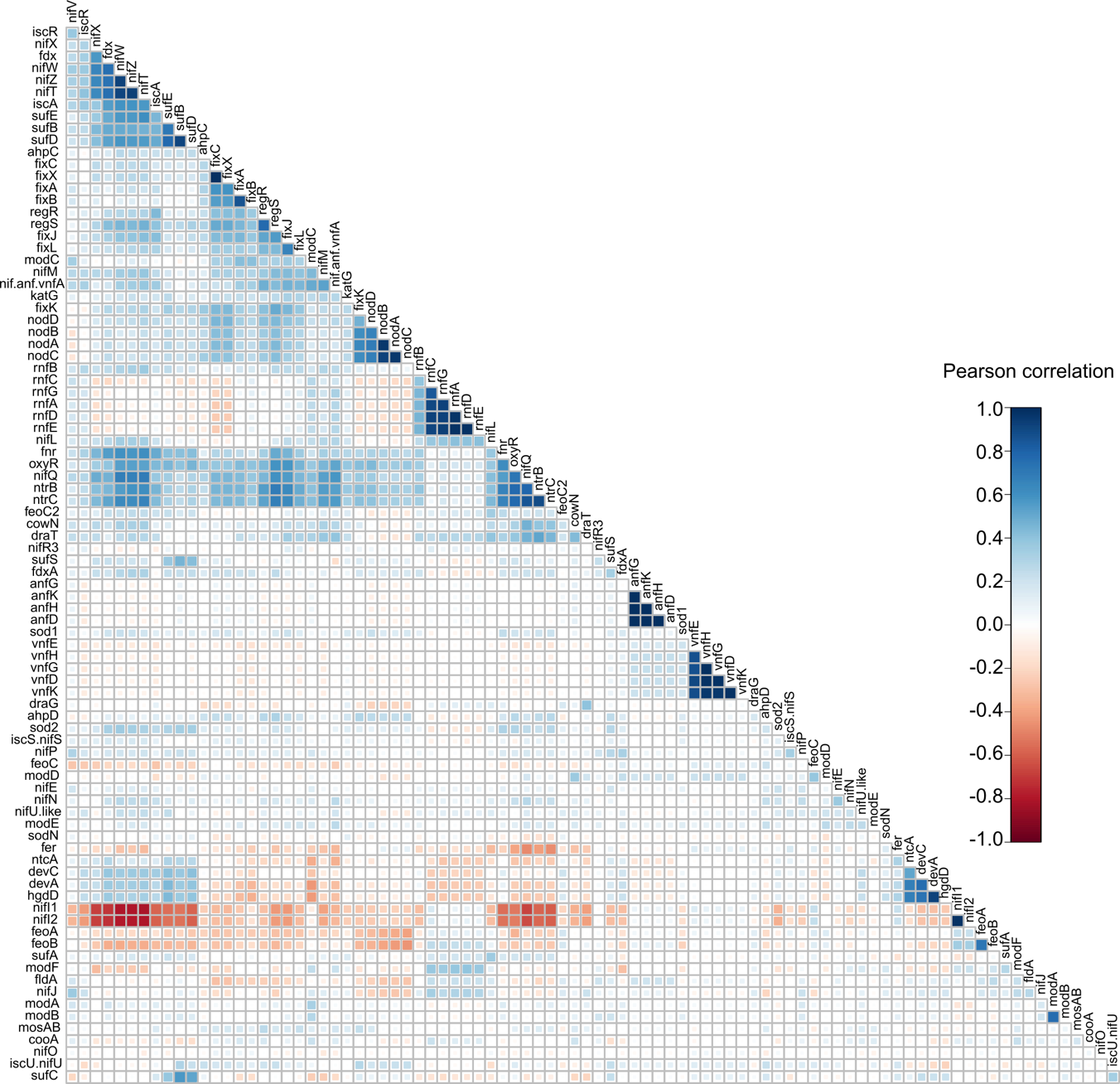


**Supplemental Figure 13.** Pearson correlation of gene co-occurrence among N_2_ fixation genes, based on the presence-absence matrix in Supplemental Figure 1. Each cell represents the correlation between a pair of genes, with blue indicating positive correlation (+1) and red indicating a negative correlation (-1). Correlations were calculated with the *cor* function in the stats R package [4] and plotted with corrplot [8] using hierarchical clustering. Non-significant correlations (p>0.05) were left blank.


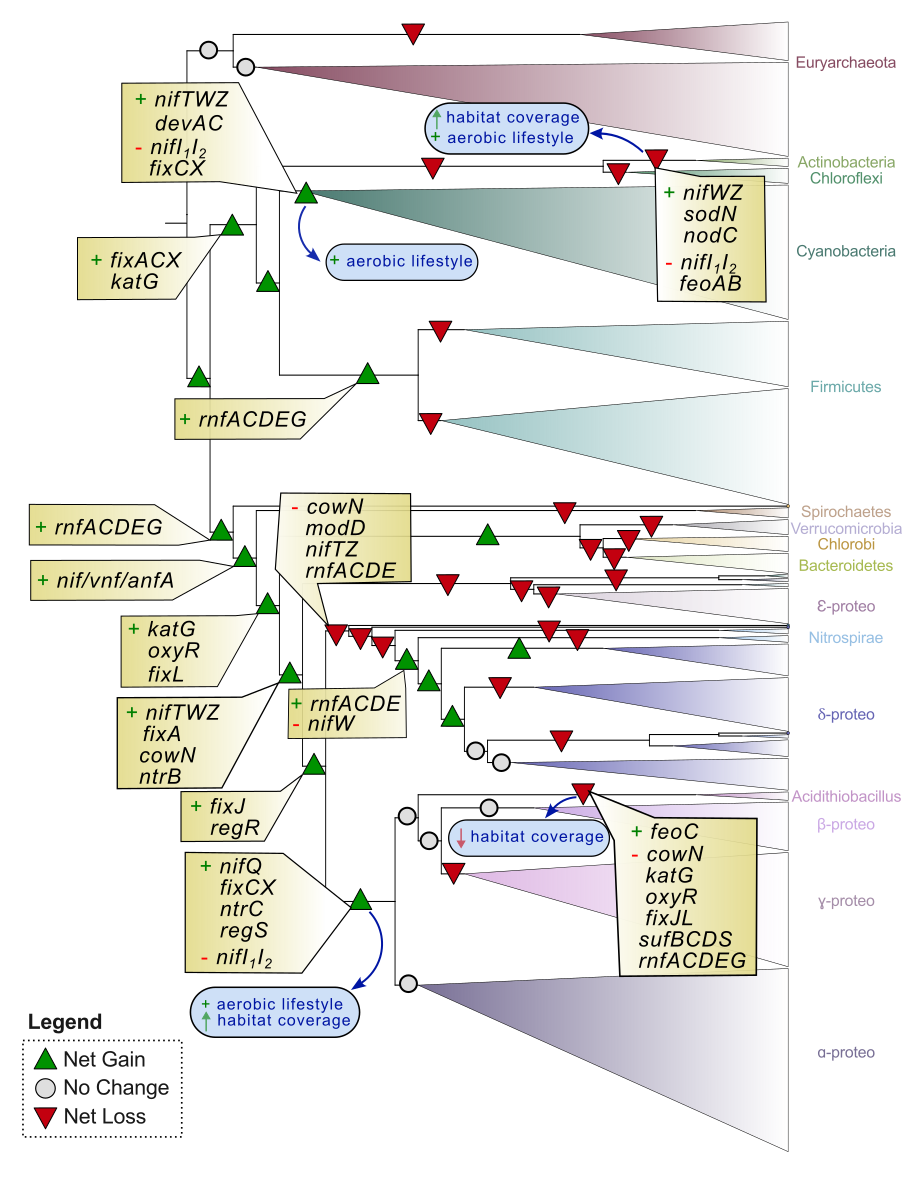


**Supplemental Figure 14.** N_2_ fixation gene gain (green triangles) and gene loss (red triangles) throughout the evolution of diazotrophs using a gain/loss penalty ratio of 1.8. Events are mapped to the WoL [1] species tree which was pruned to include only tips associated with diazotrophs identified in this study (n=673). Gene gain dominated the earliest nodes, whereas gene loss was most common near terminal branches. No change (grey circle) represents no gain or loss at that node, or an equal number or gains and losses.


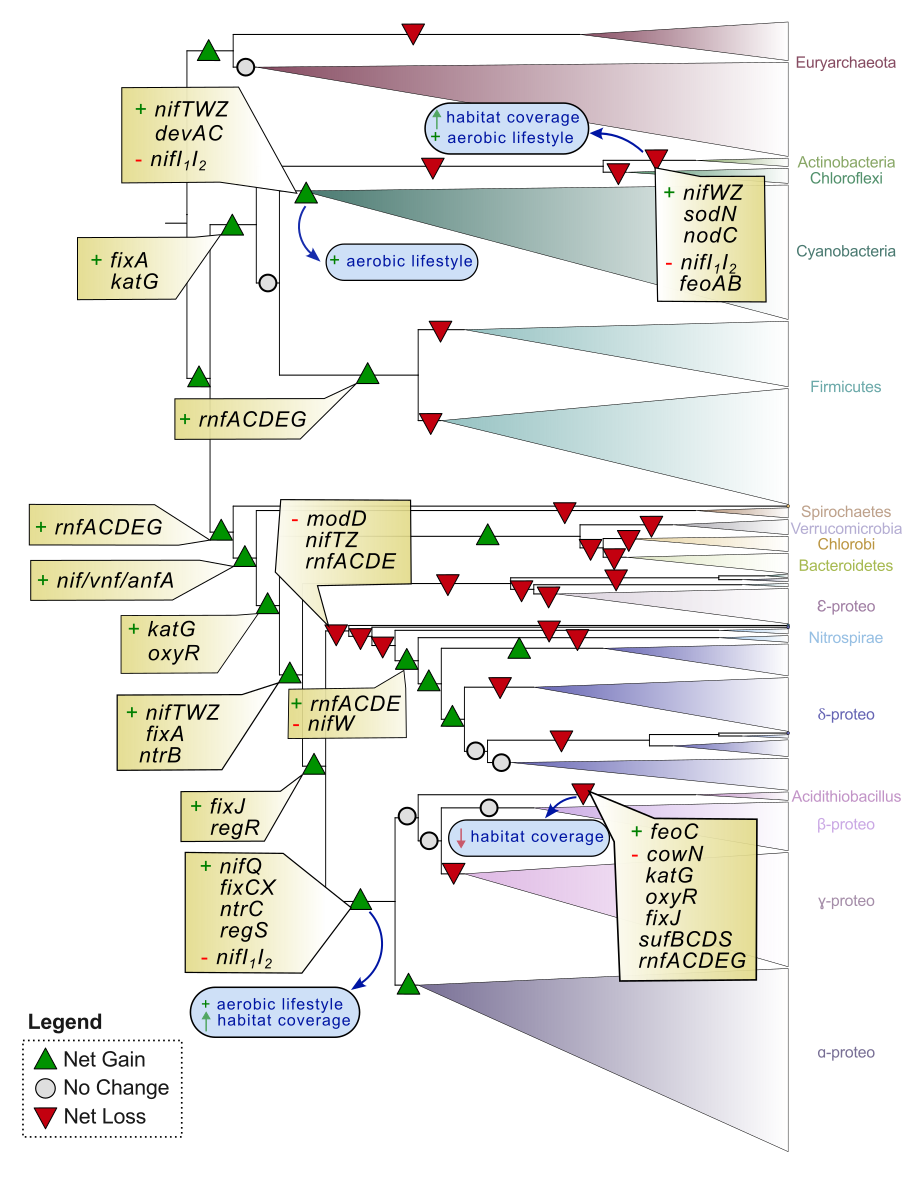


**Supplemental Figure 15.** N_2_ fixation gene gain (green triangles) and gene loss (red triangles) throughout the evolution of diazotrophs using a gain/loss penalty ratio of 1.6. Events are mapped to the WoL [1] species tree which was pruned to include only tips associated with diazotrophs identified in this study (n=673). Gene gain dominated the earliest nodes, whereas gene loss was most common near terminal branches. No change (grey circle) represents no gain or loss at that node, or an equal number or gains and losses.

**
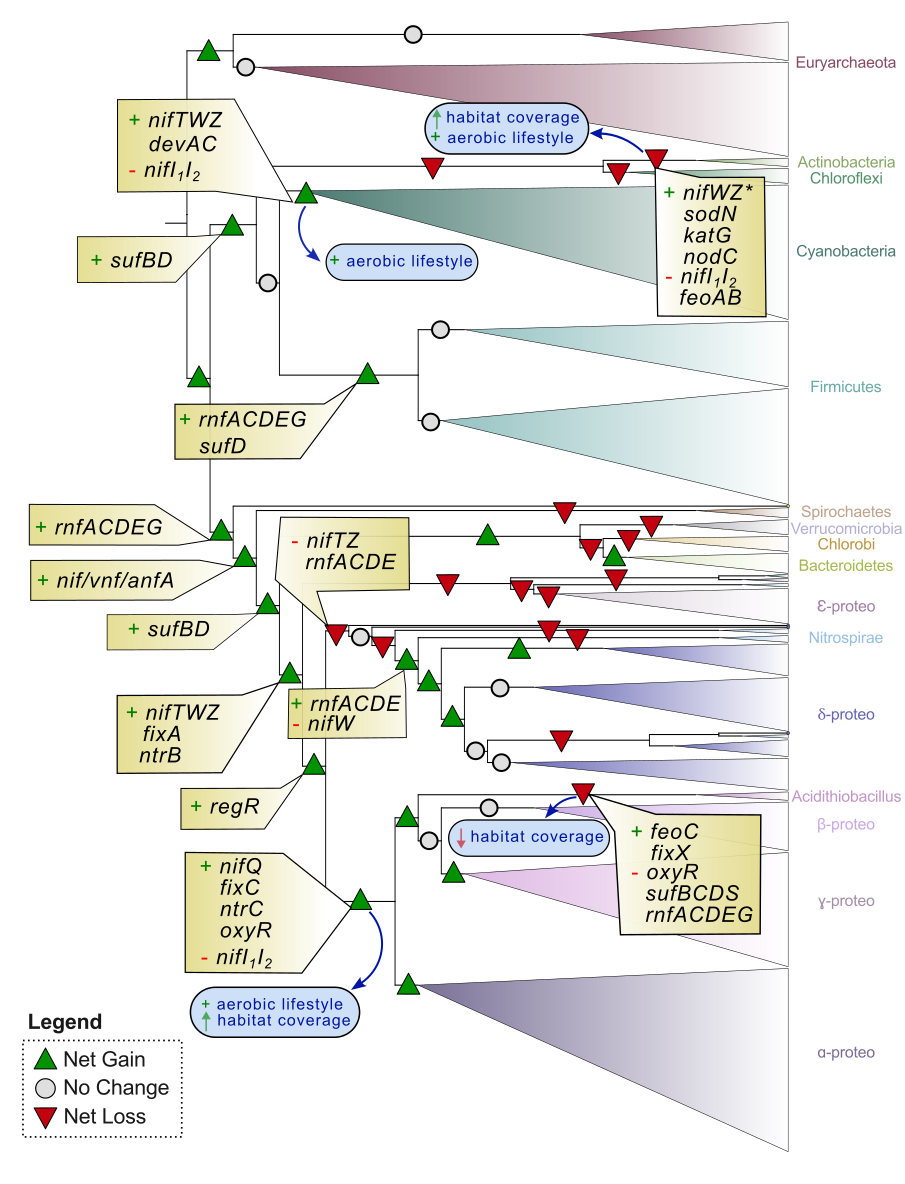
**

**Supplemental Figure 16.** N_2_ fixation gene gain (green triangles) and gene loss (red triangles) throughout the evolution of diazotrophs using a gain/loss penalty ratio of 1.4. Events are mapped to the WoL [1] species tree which was pruned to include only tips associated with diazotrophs identified in this study (n=673). Gene gain dominated the earliest nodes, whereas gene loss was most common near terminal branches. No change (grey circle) represents no gain or loss at that node, or an equal number or gains and losses.


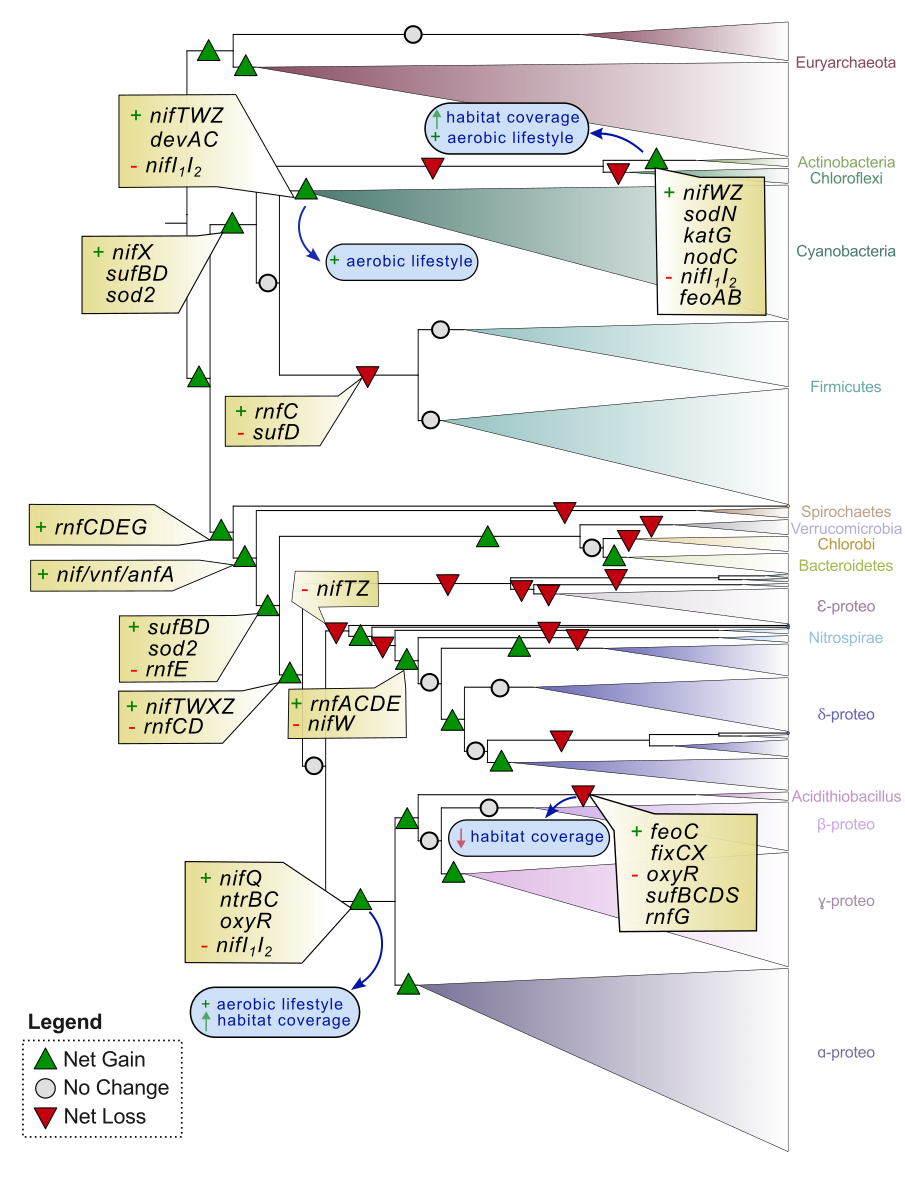


**Supplemental Figure 17.** N_2_ fixation gene gain (green triangles) and gene loss (red triangles) throughout the evolution of diazotrophs using a gain/loss penalty ratio of 1.2. Events are mapped to the WoL [1] species tree which was pruned to include only tips associated with diazotrophs identified in this study (n=673). Gene gain dominated the earliest nodes, whereas gene loss was most common near terminal branches. No change (grey circle) represents no gain or loss at that node, or an equal number or gains and losses.


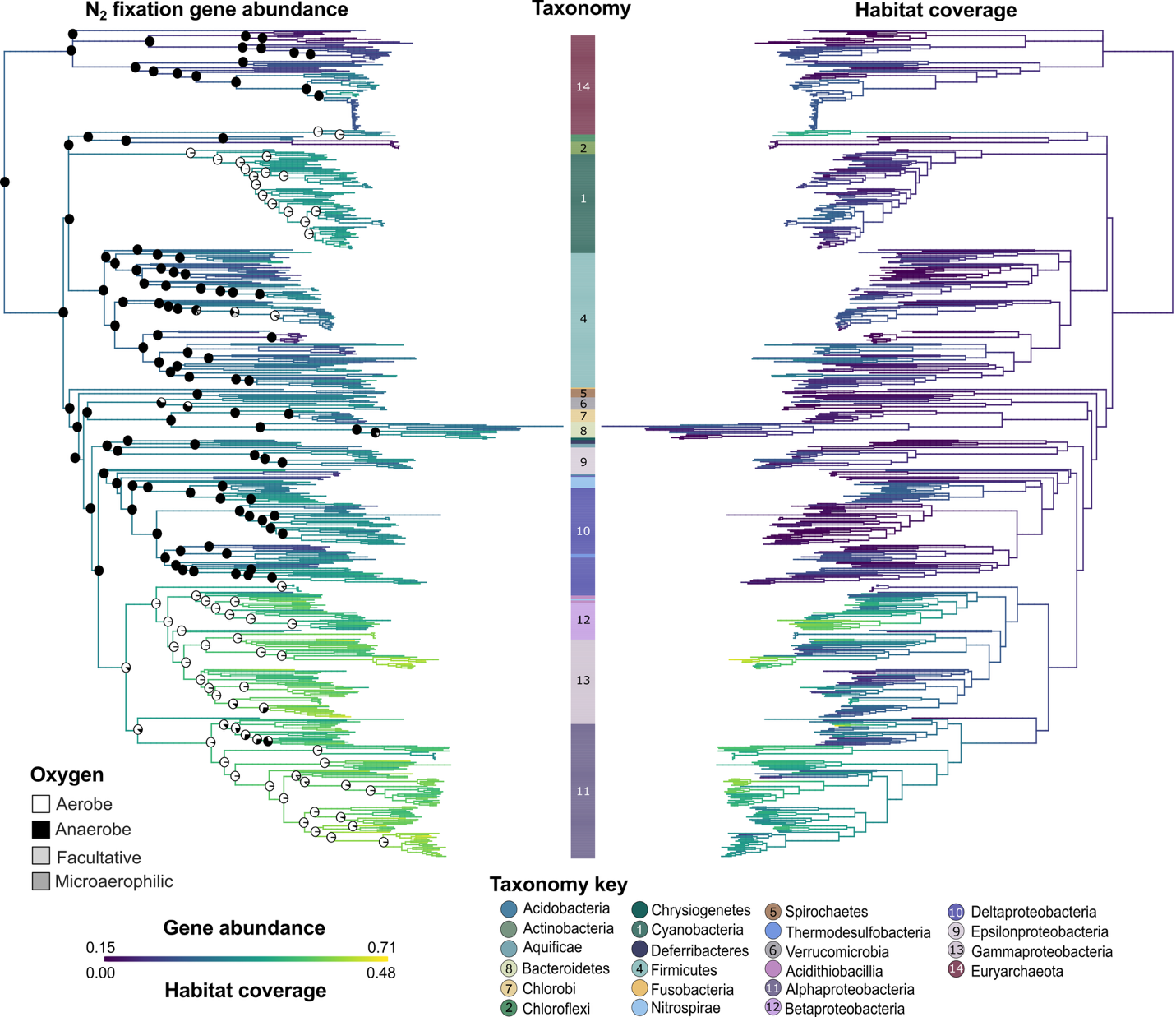


**Supplemental Figure 18.** Ancestral state reconstructions of N_2_ fixation gene abundance (right) habitat coverage (left) across the species phylogeny of nitrogenase-containing genomes. Darker branch colors represent a higher estimated gene abundance or broader habitat coverage. The *fastAnc* function in phytools was used for continuous variable reconstructions of metabolic and habitat diversity, which was mapped onto the tree using the *contMap* function [7]. On the gene abundance tree, reconstructed probabilities of ancestral oxygen preference are shown at selected nodes, with pie chart symbols representing the most probable states (aerobic, anaerobic, facultative, or microaerophilic). The *fitdiscrete* function in geiger [9] was used for discrete reconstructions of oxygen preference, which were mapped onto the phylogeny with the *simmap* function in phytools [7].

**References**

[1] Zhu Q, Mai U, Pfeiffer W, Janssen S, Asnicar F, Sanders JG, et al. Phylogenomics of 10,575 genomes reveals evolutionary proximity between domains Bacteria and Archaea. Nat Commun 2019;10:5477.

[2] Aramaki T, Blanc-Mathieu R, Endo H, Ohkubo K, Kanehisa M, Goto S, et al. KofamKOALA: KEGG Ortholog assignment based on profile HMM and adaptive score threshold. Bioinformatics 2020;36:2251–2.

[3] Yu G. Using ggtree to Visualize Data on Tree-Like Structures. Curr Protoc Bioinformatics 2020;69:e96.

[4] R Core Team. R: A Language and Environment for Statistical Computing. Vienna, Austria: R Foundation for Statistical Computing; 2014. R Foundation for Statistical Computing. 2015.

[5] Jombart T, Balloux F, Dray S. Adephylo: New tools for investigating the phylogenetic signal in biological traits. Bioinformatics 2010;26:1907–9.

[6] Villanueva RAM, Chen ZJ. ggplot2: Elegant Graphics for Data Analysis (2nd ed.). Measurement (Mahwah NJ) 2019;17:160–7.

[7] Revell LJ. phytools 2.0: an updated R ecosystem for phylogenetic comparative methods (and other things). PeerJ 2024;12:e16505.

[8] Wei T, Simko V. R package “corrplot”: Visualization of a Correlation Matrix. 2024.

[9] Pennell MW, Eastman JM, Slater GJ, Brown JW, Uyeda JC, FitzJohn RG, et al. Geiger v2.0: An expanded suite of methods for fitting macroevolutionary models to phylogenetic trees. Bioinformatics 2014;30:2216–8.
